# Supplementary material for: Clinical Origin and Species Distribution of Fusarium spp. Isolates Identified by Molecular Sequencing and Mass Spectrometry: A European Multicenter Hospital Prospective Study
Source: J Fungi (Basel). 2021 Mar 25;7(4):246. doi: 10.3390/jof7040246 (PMC8064482; doi:10.3390/jof7040246)
Supplement: Supplementary file 1 [file jof-07-00246-s001.zip › Supplemental Data Table S1.docx]

Supplementary Data Table S1: list of participating centers.

| COUNTRY | CITY | CENTER | Abbreviation | Number of *Fusarium* isolates included in the study |
| --- | --- | --- | --- | --- |
| Belgium | **ROULERS** | ROESELARE Hospital | AZR | 1 |
| Denmark | **AARHUS** | AARHUS University Hospital | AAR | 4 |
| France | **BORDEAUX** | Bordeaux Teaching Hospital | BDX | 7 |
|  | **DIJON** | Dijon Teaching Hospital | DIJ | 8 |
|  | **LILLE** | Lille Teaching Hospital | LIL | 5 |
|  | **MARSEILLE** | Timone Teaching Hospital | MAR | 8 |
|  | **NICE** | Nice Teaching Hospital | NCE | 15 |
|  | **PARIS** | Avicenne Teaching Hospital | AVC | 8 |
|  |  | Bichat Teaching Hospital | BCH | 9 |
|  |  | BIO PARIS OUEST Laboratory | BPO | 17 |
|  |  | Pitié Salpêtrière Teaching Hospital | PSL | 7 |
|  |  | Saint-Antoine Teaching Hospital | SAT | 1 |
|  | **ROUEN** | Rouen Teaching Hospital | ROU | 11 |
|  | **SAINT-ETIENNE** | Saint-Etienne Teaching Hospital | STE | 13 |
|  | **TOULOUSE** | Toulouse Teaching Hospital | TLS | 24 |
| Spain | **BARCELONA** | Barcelona Teaching Hospital | BAR | 7 |
| Sweden | **STOCKHOLM** | Karolinska University (Huddinge) | KAR | 2 |
|  |  | Karolinska University (Solna) | KAR2 | 27 |
| Switzerland | **GENEVA** | Geneva Hospital | GEN | 6 |
|  | **LUZERN** | Sylab Laboratory | LUZ | 2 |
