# Supplementary material for: Clinical Origin and Species Distribution of Fusarium spp. Isolates Identified by Molecular Sequencing and Mass Spectrometry: A European Multicenter Hospital Prospective Study
Source: J Fungi (Basel). 2021 Mar 25;7(4):246. doi: 10.3390/jof7040246 (PMC8064482; doi:10.3390/jof7040246)
Supplement: Supplementary file 1 [file jof-07-00246-s001.zip › Supplemental Data Table S2.docx]

Supplemental Data Table S2: repartition of 182 *Fusarium* sp isolates within 28 clusters of identical translation elongation factor (TEFα) sequences

| Group name | number of sequences per group | number of centers represented in the group | Isolates names |
| --- | --- | --- | --- |
| G1 | 2 | 2 | DIJ-01 / KAR2-14 |
| G2 | 2 | 1 | TLS-01 / TLS-06a |
| G3 | 4 | 4 | AVC-08 / KAR2-09 / LIL-04 / TLS-24a |
| G4 | 6 | 4 | AVC-01 / BPO-01 / BPO-12 / KAR2-27 / KAR2-29 / PSL-04 |
| G5 | 2 | 2 | KAR2-15 / LIL-01 |
| G6 | 4 | 3 | AVC-07 / BPO-02 / BPO-06 / PSL-02 |
| G7 | 2 | 2 | NCE-28 / PSL-03 |
| G8 | 13 | 7 | AVC-04 / BAR-03 / BAR-08 / BPO-03 / BPO-04 / BPO-10 / BPO-11 / BPO-16 / GEN-04 / NCE-11 / NCE-30 / ROU-22 / TLS-04 |
| G9 | 2 | 2 | BCH-05 / PSL-06 |
| G10 | 4 | 3 | DIJ-02 / DIJ-06 / ROU-16 / STE-05 |
| G11 | 2 | 1 | KAR2-07 / KAR2-18 |
| G12 | 2 | 1 | BCH-01 / BCH-02 |
| G13 | 7 | 3 | DIJ-08 / GEN-02 / KAR2-01 / KAR2-08 / KAR2-19 / KAR2-20 / KAR2-21 |
| G14 | 2 | 1 | MAR-02 / MAR-04 |
| G15 | 2 | 1 | ROU-03 / ROU-21 |
| G16 | 3 | 1 | TLS-07 / TLS-11a / TLS-12 |
| G17 | 2 | 1 | TLS-15 / TLS-18 |
| G18 | 2 | 1 | TLS-22 / TLS-23 |
| G19 | 2 | 2 | NCE-17 / TLS-08 |
| G20 | 2 | 2 | DIJ-04 / STE-03 |
| G21 | 8 | 3 | BDX-12 / KAR2-11 / KAR2-16 / KAR2-23 / TLS-10 / TLS-16a / TLS-16c / TLS-21 |
| G22 | 3 | 2 | BPO-06 / BPO-14 / NCE-36 |
| G23 | 2 | 1 | GEN-05 / GEN-06 |
| G24 | 7 | 3 | AVC-03 / AVC-05 / BAR-02 / BAR-06 / BCH-03 / BCH-08 / BCH-09 |
| G25 | 3 | 3 | BDX-09 / BPO-15 / BAR-05 |
| G26 | 2 | 1 | DIJ-03 / DIJ-07 |
| G27 | 30 | 9 | KAR2-02 / KAR2-13 / KAR2-17b / KAR2-22 / KAR-02 / LUZ-02 / MAR-01 / MAR-03 / MAR-05 / MAR-06 / MAR-08 / NCE-04 / NCE-09 / NCE-12 / NCE-15 / NCE-33 / PSL-01 / ROU-02 / ROU-05 / ROU-07 / ROU-08 / ROU-17b / STE-02 / STE-04 / STE-06 / STE-08 / STE-09 / STE-09b / STE-13 / TLS-27a |
| G28 | 2 | 2 | BPO-05 / GEN-01 |
